# XDR-*Pseudomonas aeruginosa* Outside the ICU: Is There Still Place for Colistin?

**DOI:** 10.3390/antibiotics11020193

**Published:** 2022-02-01

**Authors:** Paola Del Giacomo, Francesca Raffaelli, Angela Raffaella Losito, Barbara Fiori, Mario Tumbarello

**Affiliations:** 1 Dipartimento di Scienze di Laboratorio e Infettivologiche, Fondazione Policlinico Universitario A. Gemelli IRCCS, 00168 Rome, Italy; paola.delgiacomo@policlinicogemelli.it (P.D.G.); francesca.raffaelli@policlinicogemelli.it (F.R.); angelaraffaella.losito@policlinicogemelli.it (A.R.L.); barbara.fiori@policlinicogemelli.it (B.F.); 2 Dipartimento di Biotecnologie Mediche, Università degli Studi di Siena, 16, 53100 Siena, Italy; 3 UOC Malattie Infettive e Tropicali, Azienda Ospedaliero Universitaria Senese, 53100 Siena, Italy

**Keywords:** *Pseudomonas aeruginosa*, extensively drug resistant (XDR), ceftolozane/tazobactam, ceftazidime/avibactam, colistin

## Abstract

Background: *Pseudomonas aeruginosa* represents, among the nosocomial pathogens, one of the most serious threats, both for the severity of its clinical manifestations and its ability to develop complex profiles of resistance; Methods: we retrospectively collected the data of 21 patients admitted to a tertiary-care University Hospital of Rome with infections due to XDR-*P. aeruginosa* isolates during the second half of 2020; Results: in our institution, the percentage of XDR-*P. aeruginosa* isolates is 3.1%. None of the patients was admitted to the intensive care unit at the moment of the infection’s onset. Susceptibility to colistin was preserved in all the tested isolates. Rates of resistance to ceftolozane/tazobactam and ceftazidime/avibactam in these XDR strains were consistent; Conclusions: XDR-*P. aeruginosa* can be a threatening problem even outside the ICUs, especially in frail patients in wards with features of long-term acute care hospitals. In such a setting, ceftolozane/tazobactam and ceftazidime/avibactam should be administered with caution taking into account the microbiological susceptibility results. Colistin, even with its known safety and efficacy limits, could represent the only available therapeutic option due to its highly preserved susceptibility against XDR isolates of *P. aeruginosa*.

## 1. Introduction

*Pseudomonas aeruginosa* (PA) is a common nosocomial pathogen worldwide and it represents one of the most challenging microorganisms to face in clinical practice due to its intrinsic resistance and its extraordinary ability to develop additional resistance through selection of chromosomal mutations and acquisition of resistance genes [1]. The isolation of extensively drug-resistant *Pseudomonas aeruginosa* strains (XDR-PA) seriously compromises the probability of receiving an appropriate initial antibiotic therapy, possibly leading to poor outcomes, particularly in the presence of severe infections.

## 2. Methods

We retrospectively collected, through the daily microbiological report of multidrug-resistant (MDR) or extensively drug-resistant (XDR) isolates delivered by the Microbiology Laboratory, data of 21 patients admitted to a tertiary-care at the University Hospital of Rome (Fondazione Policlinico Universitario Agostino Gemelli IRCCS) with documented XDR-*Pseudomonas aeruginosa* isolates from 1 June to 31 December 2020. Patients were selected if they had documented true infections due to *Pseudomonas aeruginosa* strains non-susceptible to at least one agent in all but two or fewer antimicrobial categories according to the established definition of XDR (anti-pseudomonal cephalosporins, anti-pseudomonal penicillin plus β-lactamase inhibitors, monobactams, anti-pseudomonal carbapenems, aminoglycosides, fluoroquinolones, phosphonic acid and polymyxins) [2]. Each patient was included only once, at the time of the XDR-PA infection onset. Infections were classified as XDR-PA bacteremia if blood cultures were positive for XDR-PA strain (with or without the same XDR-PA isolation from other sites) and there were clinical signs of systemic inflammatory response syndrome.

Two patients were excluded because they were not classified as infections but colonizations. Colonization was defined as the isolation of PA from any clinical specimens (except blood) in the absence of signs or symptoms of infections, and the patients had not received antimicrobial treatment related to the culture results.

Isolates were identified using a MALDI Biotyper (Bruker Daltonik GmbH, Leipzig, Germany).

The minimum inhibitory concentrations (MICs) of ceftazidime, cefepime, ceftazidime/avibactam, ceftolozane/tazobactam, piperacillin-tazobactam, meropenem, imipenem, ciprofloxacin, amikacin, gentamicin and tobramycin were determined by a VITEK^®^2 system (bioMérieux) and, for colistin, when feasible, by commercial broth-microdilution antimicrobial susceptibility testing panels (MERLIN Diagnostica GmbH, Bornheim, Germany) according to EUCAST guidelines. Results were interpreted according to European Committee on Antimicrobial Susceptibility Testing (EUCAST) clinical breakpoints [3,4]. The NG-Test Carba 5 immunochromatographic assay (NG Biotech, Guipry, France) was used to detect the five main carbapenemases, i.e., KPC, OXA-48-like, NDM, VIM, and IMP [5].

Adequate empiric therapy was deemed an appropriate dosage regimen of one or more antibiotics to which the isolate was sensitive in vitro. An effective definitive therapy was defined as treatment with at least one effective anti-pseudomonal agent based on antimicrobial susceptibility testing. The timing to evaluate the appropriate initiation of a target antibiotic therapy was set at 48 h from the infection onset.

Clinical success was defined as the resolution of signs and symptoms of acute infection. Absence of recurrent infection was evaluated at 30 days following the onset of infection or at time of discharge from the hospital.

Microbiological failure was defined as isolation of *Pseudomonas aeruginosa* with the same susceptibility profile at the same site ≥7 days after the target therapy initiation.

## 3. Results

Nineteen patients with XDR-PA infections were included in the study. The median age was 69 (IQR 64–80). Males comprised 68.4%. No community-acquired infection was found; 11 (57.9%) of the infections were classified as hospital-acquired infection and were inpatients belonging to medical wards at the time of the infection onset: 63.6% of the patients in the Pneumology and the Neurology/Neurological rehabilitation clinic. Eight (42.1%) of the patients were assumed to have a healthcare-related infection. In five (26.3%) cases, an admission in an Intensive Care Unit (ICU) in the previous 90 days was reported. Eight (42.1%) patients had undergone surgical or invasive procedures in the previous 30 days. All the patients had at least one comorbidity, with a median Age-Adjusted Charlson Comorbidity index of 6.

In 16 (84.2%) patients, a previous bacterial infection was documented. Among these, a previous PA infection was documented in six (31.6%) patients. Prior isolation of MDR/XDR bacteria was reported in 11 (57.9%) patients (Methicillin-resistant *Staphylococcus aureus*, Vancomycin-resistant *Enterococcus*, Carbapenem-resistant *Enterobacterales*, MDR/XDR-PA). Previous antibiotic therapy was reported in 15 (78.9%) patients. Carbapenems were the most frequent prior antimicrobials reported (42.1%), together with glycopeptides (42.1%) and followed by piperacillin/tazobactam (36.8%). In 14 (73.7%) patients, prior antimicrobial use was as long as seven days or longer. In 12 (63.2%) cases, the antimicrobial therapy consisted of multiple antibiotics (sequential or used in combinations). One or more invasive medical devices were present in 17 (89.5%) of the patients: Foley catheter was present in 14 (73.7%), tracheostomy in seven (36.8%) of the patients, central venous catheter in five of the patients (26.3%).

True XDR-PA infections included, six (31.6%) UTIs, five (26.3%) BSIs, four (21.1%) pneumonia (2 HAPs, 2 VAPs), three (15.8%) ABSSSIs and one (5.3%) PJI. Infections due to XDR-PA were polymicrobial in 6 (31.6%) cases.

Septic shock was reported in 21.1% of the patients in the medical report.

Demographic and clinical characteristics of the patients selected are reported in Table 1.

Antimicrobial susceptibility of the tested isolates is listed in Table 2. Notably, all the isolates tested for colistin were susceptible. All the isolates were tested for ceftazidime/avibactam (C/A) and ceftolozane/tazobactam (C/T). Eight (42.1%) were reported as susceptible to C/A and seven (36.8%) to C/T. Among the aminoglycoside class, six (42.9%) out of 14 isolates tested for amikacin were susceptible, while none of the 11 isolates tested for gentamicin was susceptible, three out of eight isolates tested for tobramycin were susceptible. Two out of 19 (10.5%) tested isolates were susceptible to piperacillin/tazobactam and six out of 19 (31.6%) to meropenem.

As reported in Table 3**,** empiric therapy was administered in 14 (73.7%) of the patients, but it was appropriate only in two (14.3%) cases. The time to start a target antibiotic therapy exceeded 48 h in 15 (78.9%) cases. A targeted treatment consisted of anti-pseudomonal monotherapy in 12 (63.2%) of the cases. In six cases, colistin monotherapy was administered based on antimicrobial susceptibility data, and in two of them, inhaled colistin was associated with pneumonia. The median duration of therapy was 10 days (IQR 7–14). Source control was performed in eight (42.1%) of the cases. Clinical success was achieved in 17 (89.5%) of the patients. Microbiologic failure was evaluated in 13 patients out of 19 through repeated cultures at the same site after at least seven days from the initiation of effective antibiotic therapy and reported in three patients (23.1%). Two of them experimented clinical success later on, the other died due to a recurrent infection within 30 days.

One patient died due to a succeeding *Candida* bloodstream infection.

## 4. Discussion

Combined resistance to five antimicrobial groups is reported in PA around 3.4% in Europe [6]. Fewer data exist on the prevalence of XDR-PA [7,8]. Surprisingly, one Spanish survey reported that 17% of PA infections was due to XDR strains [1].

In our institution, the percentage of XDR-PA isolates on the total of the *Pseudomonas aeruginosa* isolates (n.609) from all sources from 1 June to 31 December 2020 is 3.1%. This epidemiological result is in line with other previous reports [9].

Risk factors for XDR-PA have been exhaustively investigated in several studies and mostly addressed in prior hospital stay, previous antibiotic treatment (especially carbapenems and fluoroquinolones), prior ICU stay, previous PA colonization or infection, multiple comorbidities, and indwelling urinary catheter [10,11,12,13,14,15,16,17].

In our report, the majority of the patients had a median Age-Adjusted Charlson Comorbidity Index of 6 with at least one comorbidity, mostly chronic lung disease, diabetes, solid tumors, and chronic kidney disease. Among five patients with a previous PA infection/colonization, two were MDR/XDR (1/1, respectively). Previous PA isolation was consistent in our cohort, suggesting that a PA strain can build up resistance sequentially in the same patient.

Curiously, no patient was admitted to ICU at the time of the infection onset, even if 26.3% had reported a previous ICU stay. The infections developed mainly in medical wards such as Pneumology or high-intensity rehabilitation, where patients were often exposed to a unique combination of hazardous conditions for infections. A history of prior use of antibiotics was reported in the majority of the patients (78.9%), with carbapenems (42.1%) as the most frequent drug prescribed. The previous use of multiple antibiotics (in combinations or sequential over time) was present in 63.2% of the patients’ history, possibly reflecting the importance of antimicrobial pressure on the development of resistance in PA.

For XDR-PA, polymyxin B, colistin, C/T, C/A, aztreonam, aminoglycosides and, recently, cefiderocol are the main therapeutic options depending on resistance profile. Combination therapy is not currently recommended. Treatment-emergent mutations conferring resistance to the new compounds are already reported [18,19,20,21,22,23].

In our case series, colistin was the only antibiotic to which 100% of tested isolates were susceptible. Further, we observed that 26.3% of our isolates were susceptible only to colistin. These data agree with others already reported in Southern European countries’ literature, suggesting that colistin could represent the only therapeutic option available in these patients, although with some pharmacological and safety concerns [1,24,25,26,27].

Resistance to C/T and/or C/A is significant among the XDR-PA isolates selected. In literature, resistance is reported mainly due to carbapenemase production and some AmpC mutants. Other potential resistance mutations include specific large chromosomal deletions and PBP3 mutations, reduced membrane permeability and efflux pumps [20,28,29,30,31].

Even if the overall susceptibility rates of C/T against PA are high (94.1% in Western Europe and 80.9% in Eastern Europe, respectively), the retained activity of this compound against meropenem non-susceptible PA isolates drops to 75.2% and 59.2% in Western and Eastern Europe, respectively. Even lower rates have been reported from Southern Asia (37.9%) [30,32]. Low susceptibility rates to C/T in the subgroup of XDR-PA isolates have already been reported in Europe and the Middle East literature [33,34,35].

In our case series, probably due to the high percentage of resistance, C/T has been rarely used in target therapy. In four cases, even if susceptibility to C/T was documented, meropenem was chosen as target therapy or continued if started as empiric therapy due to clinical improvement without an Infectious Disease specialist’s consultation. In another case due to a strain susceptible to C/T, a UTI, amikacin was preferred to C/T as target therapy due to the possibility of a single dose administration in a patient with serious difficulty in having stable venous access. In one case, C/A was adopted as a target therapy for a polymicrobial infection due to XDR-PA and *Klebsiella pneumoniae* carbapenemase-producing *K. pneumoniae*.

In all but two cases, C/T resistance was shared with C/A. The cross-resistance between the two compounds has already been documented [36].

Among the 19 isolates, those belonging to blood cultures (n.5) were resistant to both C/A and C/T. All these isolates were tested retrospectively for the presence of carbapenemase enzymes and two out of five were found to harbor a carbapenemase VIM type. One respiratory tract sample PA-isolate, resistant to both the new drugs, was also evaluated for carbapenemases. An IMP-type carbapenemase was found. This finding of the prevalence of MBL in PA isolates has already been underlined in previous reports [37,38] and highlights that production of MBL carbapenemases is one of the main determinants leading to complex resistance profiles. While the development of resistance to C/T and C/A during treatment of PA infections is already concerning, in our case series the development of resistance to C/T and C/A was apparently not related to previous exposure to these drugs with the exception of one patient who had a recent history of exposure to both drugs at the time of the infection onset [23].

Consistent with other reports, among aminoglycosides, our data reveal that PA shows a higher susceptibility for amikacin then for other aminoglycosides (tobramycin and gentamicin) [39,40,41].

Even if empiric therapy was prescribed to the majority of the patients with XDR-PA infections in our case series it was rarely appropriate and a target therapy was usually not administered in the first 48 h. In case of XDR profile it is highly unlikely that an appropriate empiric monotherapy can be chosen without knowledge of the isolate susceptibility.

Resistance in PA isolates has already been reported in other studies as an independent factor for an inappropriate initial antibiotic treatment, resulting in poor outcomes and increased costs. The antimicrobial susceptibility pattern of the infecting strain is obviously not available when empirical therapy is prescribed and an XDR profile is not usually taken into account outside the ICU and in patients who are not critically ill [42,43,44,45,46,47,48,49].

Due to the retrospective design of our study, the very small sample size and the inclusion of patients with different types of infections, no conclusive results about mortality can be deduced. However, it is remarkable that three (23.1%) of the patients evaluated for microbiological failure with control cultures at the same site experienced the re-isolation of PA with the same resistance profile. Respiratory infections were found in 2/3 of the cases in which microbiological eradication was not achieved. One of these patients experienced a recurrent XDR-PA infection with a fatal outcome. The worse outcome of lung infections has already been underlined, probably due to pharmacokinetic concerns of available drugs, infeasibility of adequate source control and, sometimes, more critical conditions [50].

## 5. Conclusions

Our data show that XDR-PA can be a threatening problem even outside the ICUs, especially in some wards that simulate the picture of long-term acute care hospitals: facilities specialized in the treatment of patients with serious medical conditions that no longer need intensive care but require more than they can receive in a rehabilitation center. Such frail patients can be more prone to acquire XDR-PA due to a combination of risky conditions (long hospital or previous ICU stay, multiple medical devices, previous multiple antibiotic treatments, particularly carbapenems, MDR colonization).

Despite their high susceptibility and good activity in PA, even MDR, the use of C/A and C/T in the contest of some XDR-PA strains could be seriously restricted due to reduced susceptibility.

Colistin, with highly preserved susceptibility, could often represent the only available therapeutic option against such isolates, but with significant safety concerns (kidney failure) and known efficacy limits, especially in some difficult-to-treat sources of infections (i.e., lung).

Thus, in settings where resistance to C/T or C/A is present, given the well-known colistin toxicity concern, cefiderocol could become an interesting perspective in the treatment of such XDR-PA infections.

Knowing local resistance rates and geographical variation in new drugs’ susceptibility due to the distinct local molecular epidemiology is essential in approaching XDR-PA severe infections.

Further, it seems crucial to have susceptibility testing results for all newer agents available as soon as possible.

## Figures and Tables

**Table 1:** Demographic and clinical characteristics.

Characteristics	Value (*n* = 19)
Age in years, median (IQR)	69 (64–80)
Male, *n* (%)	13 (68.4)
Context of acquisition, *n* (%)	
Hospital acquired	11 (57.9)
Healthcare-related	8 (42.1)
Underlying diseases, *n* (%)	19 (100)
Charlson comorbidity index, median (range)	6 (2–8)
Chronic lung disease	6 (31.6)
Solid malignancy	5 (26.3)
Diabetes mellitus	5 (26.3)
Chronic kidney disease	4 (21.1)
Immunosuppression	3 (15.8)
Trauma	3 (15.8)
Hemodyalisis	1 (5.3)
Prior surgery within 30 days, *n* (%)	3 (15.8)
Prior invasive procedures within 30 days, *n* (%)	5 (26.3)
Prior ICU stays within 90 days, *n* (%)	5 (26.3)
Prior bacterial infections, *n* (%)	16 (84.2)
Prior PA infections within 3 months	5 (26.3)
Prior MDR/XDR pathogens, *n* (%)	11 (57.9)
Prior antimicrobial use, within 90 days *n* (%)	15 (78.9)
Carbapenem	8 (42.1)
Glycopeptide	8 (42.1)
Piperacillin/tazobactam	7 (36.8)
Cephalosporin	5 (26.3)
Colistin	3 (15.8)
Fluoroquinolone	3 (15.8)
Aminoglycoside	2 (10.5)
Ceftazidime/avibactam	1 (5.3)
Ceftolozane/tazobactam	1 (5.3)
Other	8 (42.1)
Sequential or combination use, *n* (%)	12 (63.2)
Length of prior antimicrobial use ≥7 days	14 (73.7)
Indwelling device at infection onset, *n* (%)	17 (89.5)
Urinary catheter	14 (73.7)
Tracheostomy	7 (36.8)
Central venous catheter	5 (26.3)
Polymicrobial infection, *n* (%)	6 (31.6)
Infection types, *n* (%)	
UTI	6 (31.6)
BSI	5 (26.3)
ABSSSI	3 (15.8)
HAP	2 (10.5)
VAP	2 (10.5)
PJI	1 (5.3)

*Pseudomonas aeruginosa*, PA; Multidrug-resistant, MDR; ICU, intensive care unit; urinary tract infection, UTI; bloodstream infections, BSI; Hospital-Acquired Pneumonia, HAP; Ventilator-Associated Pneumonia, VAP; acute bacterial skin and skin structure infections, ABSSIs; prosthetic joint infection, PJI.

**Table 2:** Rates of antimicrobials’ susceptibility.

Antibiotics	No. Isolates Tested	Susceptibility *n* (%)
Ceftazidime	19	3 (15.8)
Cefepime	19	1 (5.3)
Ceftazidime/avibactam	19	8 (42.1)
Ceftolozane/tazobactam	19	7 (36.8)
Piperacillin/tazobactam	19	2 (10.5)
Meropenem	19	6 (31.6)
Imipenem	11	3 (27.3)
Ciprofloxacin	19	1 (5.3)
Colistin	13	13 (100)
Gentamicin	11	0 (0)
Amikacin	14	6 (42.9)
Tobramicin	8	3 (37.5)

**Table 3:** Antibiotic therapies and outcomes.

Antibiotic Therapies and Outcome	Value (*n* = 19)
Empiric therapy, *n* (%)	14 (73.7)
Appropriate empiric therapy, *n* (%)	2 (10.5)
Start of a target therapy >48 h, *n* (%)	15 (78.9)
**Target monotherapy, *n* (%)**	12 (63.2)
Colistin	6 (31.2)
Meropenem	3 (15.8)
Piperacillin/tazobactam	1 (5.3)
Amikacin	1 (5.3)
Ceftazidime/avibactam	1 (5.3)
**Target Combination therapy, *n* (%)**	7 (36.8)
Ceftazidime/Avibactam + Colistin	2 (15.4)
Colistin + Meropenem	2 (15.4)
Ceftolozane/tazobactam + Amikacin	1 (5.3)
Ceftazidime/avibactam + Amikacin	1 (5.3)
Colistin + Piperacillin/tazobactam	1 (5.3)
Source control, *n* (%)	8 (42.1)
Length of therapy in days, median (IQR)	10 (7–14)
Death (30 days), *n* (%)	2 (15.4)
Clinical success, *n* (%)	17 (89.5)
Microbiological failure, *n*/patients evaluated (%)	3/13 (23.1)
Recurrent infection, *n* (%)	1 (5.3)

## Data Availability

The data presented in this study are available on request from the corresponding author.
